# AI in Long-Term Care: A Closed-Loop Sociotechnical Reliability Framework

**DOI:** 10.2196/105161

**Published:** 2026-10-28

**Authors:** Kyoung-Bok Min, Jin-Young Min

**Affiliations:** 1 Department of Preventive Medicine Seoul National University College of Medicine Seoul Republic of Korea; 2 Integrated Major in Innovative Medical Science Seoul National University Graduate School Seoul Republic of Korea; 3 Institute of Health and Environment Kongju National University Gonju-si, Chungcheongnam-do Republic of Korea

**Keywords:** long-term care, artificial intelligence, AI, sociotechnical reliability, sociotechnical systems, governance, human oversight

## Abstract

AI is increasingly used in long-term care (LTC), yet reliability is often judged at the level of individual models or devices. This is insufficient in LTC, where residents vary in capacity, autonomy, and preferences. Their ability to question AI outputs may fluctuate, and care delivery depends on distributed roles and finite operational capacity. We define sociotechnical reliability as the capacity of an AI-enabled care system to consistently translate resident-relevant information into appropriate and timely care actions under real-world LTC conditions, with failures that are detectable and correctable across the care pathway. In this Viewpoint, we draw on professional experience overseeing AI initiatives across LTC and hospital settings, examined in relation to a purposively mapped interdisciplinary literature, to inform the development of a reliability framework. Our aim is to reframe AI reliability in LTC as an end-to-end care system property and to provide a framework for identifying where reliability is gained, lost, and evaluated. We propose 5 reliability functions: sensing, inference, verification and contextualization, response and escalation, and learning and adaptation, with governance and accountability operating across all 5 functions. The framework highlights recurrent failures, including incomplete sensing, nonportable inference, overreliance on or dismissal of AI outputs during verification, unclear ownership or delayed response, and repeated failures without correction. The framework also makes reliability empirically examinable through questions about where the pathway fails, who has the authority to verify and act, whether response demands are sustainable within available capacity, and whether failures become visible to governance and inform learning and adaptation. Our central viewpoint is that AI in LTC should be judged not by model performance alone but by whether the surrounding care system can reliably translate AI-generated information into care.

## Introduction

AI is increasingly positioned as a response to the safety, workforce, and continuity-of-care challenges facing long-term care (LTC). Passive sensors, fall detection systems, predictive alerts, decision support tools, caregiver-facing applications, and care robots are promoted as ways of monitoring risk and augmenting care for older adults with complex functional, cognitive, and clinical needs [1,2]. In this setting, missed events or delayed recognition can translate directly into harm. Residents also differ widely in their capacity to notice, question, or contest AI outputs, and this capacity may fluctuate over time. The burden of reliability therefore cannot rest on the resident or the algorithm alone but must be carried by the surrounding care system.

Existing reviews have organized the field largely by technology type, targeted outcomes, implementation barriers, or responsible innovation principles [3-6], leaving the evidence base siloed by technology rather than integrated along the care pathway. The consequence is that each component can appear to work while the care system as a whole remains unreliable: a fall detector with high algorithmic accuracy is not a safety net if verification, escalation, and learning fail. Evidence from adjacent domains similarly indicates that consequential failures may arise at the interfaces between data capture, inference, human interpretation, workflow, response, and oversight rather than within algorithms alone [7-9].

In this Viewpoint, we propose sociotechnical reliability as an organizing concept for AI in LTC, defined as the capacity of an AI-enabled care system to consistently translate resident-relevant information into appropriate and timely care actions under real-world LTC conditions, with failures that are detectable and correctable across the care pathway. Our aim is to develop and delimit a closed-loop reliability framework that identifies where this pathway can fail, clarifies how governance and accountability operate across it, and provides an empirically examinable basis for evaluating AI-enabled care beyond model performance alone.

## Developing a Closed-Loop Reliability Framework

This Viewpoint was grounded initially in practice rather than derived from the literature. The 5 reliability functions were formulated before the literature was mapped and were informed by the corresponding author’s professional experience overseeing AI initiatives across LTC and hospital settings. A recurring concern was not simply whether an algorithm performed adequately but whether the surrounding care process could reliably interpret and act on its outputs. We subsequently examined literature across 6 domains to determine whether analogous concerns were reported more broadly, to refine the 5 functions, and to characterize the recurrent failure modes and reliability conditions associated with them.

The 6 domains were selected because each addressed a complementary component of the end-to-end reliability problem that could not be captured adequately by LTC AI literature alone. AI in LTC defined the target care context [1,4-6,10-13]; smart home technologies, wearable sensors, and ambient assisted living informed data capture, continuity, and sensing reliability [14-23]; clinical decision support and real-world clinical AI informed inference, human verification, alerting, workflow integration, and response [24-33]; and care robotics and social robots informed human-robot interaction, agency, trust, and relational consequences in older adult care [34-40]. Sociotechnical and implementation frameworks provided the work system, organizational, and adaptive foundations of the framework [8,9,41-50], while responsible AI scholarship informed fairness, transparency, privacy, accountability, and lifecycle oversight [24,51-60]. These domains were complementary rather than intended to provide an exhaustive taxonomy of relevant literature.

We used structured PubMed (MEDLINE) searches, reference-list checking, and selective forward citation checking to identify literature relevant to these functions and associated failure points. References were retained for framework mapping when they (1) addressed reliability, failure, safety, or implementation in LTC or a conceptually transferable care setting and (2) supported, challenged, qualified, or exposed gaps in 1 or more reliability functions, failure modes, or reliability conditions. Mapping was conducted by the first author, who was not involved in the initial formulation of the 5 reliability functions, and reviewed by the corresponding author, with ambiguous mappings resolved through discussion. This was not independent duplicate screening or coding. The literature process was purposive rather than systematic: it was intended to interrogate and refine a practice-informed framework against interdisciplinary literature, not to provide exhaustive coverage of the field. Using a conceptual framework to organize heterogeneous evidence has methodological precedent [61,62], although the framework in this Viewpoint originated in practice rather than from a previously published model.

During refinement, candidate reliability conditions were considered at the system level rather than by citation counting alone. A condition was retained when it addressed a recognizable failure point in translating AI-generated information into care, was recurrently supported by the relevant literature or grounded in an established sociotechnical or responsible AI principle, and could be expressed as a requirement applicable beyond a single technology or use case. No numerical citation threshold or vote counting rule was applied. Detailed search concepts, relevance criteria, and evidence mappings are provided in Multimedia Appendix 1[63-66].

To clarify the framework’s incremental contribution, Table 1 compares the proposed framework’s organizing emphasis with those of Systems Engineering Initiative for Patient Safety (SEIPS); nonadoption, abandonment, scale-up, spread, and sustainability (NASSS); and the SALIENT framework, a 5-stage framework for end-to-end implementation of clinical AI [33]. The comparison concerns each framework’s primary organizing emphasis rather than the presence or absence of individual concepts.

**Table 1:** Comparison of the proposed framework with Systems Engineering Initiative for Patient Safety (SEIPS); nonadoption, abandonment, scale-up, spread, and sustainability (NASSS); and a 5-stage framework for end-to-end deployment of clinical AI (SALIENT framework) across 5 dimensions of care system reliability^a^.

Dimensions	SEIPS 2.0/3.0 [42,43,45]	NASSS [8,41]	SALIENT framework [33]	Closed-loop reliability framework (this Viewpoint)
Resident context and variable agency	Incorporates person-centered work system and patient journey context across settings	Addresses the condition, adopters, and organizational context within technology complexity	Considers the patient population within clinical AI implementation	Treats variation in resident capacity and agency as a design condition
Signal-to-care-action pathway	Links work system configuration to outcomes but does not define an AI-specific signal pathway	Organizes complexity across technology adoption and sustainability over time	Defines a staged pathway from clinical AI development to postdeployment monitoring	Traces resident-relevant signals from capture to care action and feedback through 5 reliability functions
Verification and escalation	Models human-technology interaction and work roles	Addresses user adoption and organizational work	Incorporates human interaction, alerting, and workflow integration	Defines verification and escalation as explicit reliability functions with designated authority
Resource-aware operational feasibility	Addresses workload, staffing, tasks, and environmental influences on performance	Explicitly addresses organizational capacity and wider system complexity	Addresses implementation resources, workflow fit, and sustainability	Requires verification and response demands to match sustainable operational capacity
Distributed accountability	Represents roles and organizational structures	Addresses wider system domain including policy, regulation, and professional actors	Addresses implementation and monitoring responsibilities across stakeholders	Requires prospective assignment of operational responsibility across roles, with governance spanning all 5 functions

^a^Frameworks are compared by their primary organizing emphasis, not ranked; each contains concepts relevant to reliability. Normalization process theory and resilience engineering also inform the proposed framework but are not included in the table because neither was developed specifically as a technology deployment framework.

Existing frameworks contain several elements relevant to reliability but organize them around different primary problems. The proposed framework instead foregrounds the end-to-end reliability of a resident-relevant AI signal as it is verified and translated into care action and as subsequent outcomes feed into learning and adaptation under the operational constraints of LTC.

This organization makes 4 evaluation questions explicit: at which reliability function is the pathway failing; who holds the authority to verify the output and act on it; can the required response be sustained within available staffing and workflow capacity; and do failures become visible to governance and feed into learning and adaptation?

## From Algorithmic Performance to Care System Reliability

The aforementioned definition locates reliability in the care system rather than in any single component and defines the successful translation of AI outputs into care action, rather than output accuracy alone, as the operational criterion for reliability. Sociotechnical reliability is distinct from several adjacent concepts. Algorithmic reliability concerns the consistency and technical performance of a model or device and is therefore 1 component of, but not equivalent to, care system reliability [7,48]. Resident safety and quality of care are outcomes of care system performance rather than measures of reliability itself [42]. Resilience refers to the capacity to adapt, recover, and maintain function under disturbance and contributes particularly to learning and adaptation [44]. Implementation success concerns whether a technology is adopted, integrated, and sustained; an implemented system may nevertheless remain unreliable if verification or response fails [8,46].

Trustworthy AI provides a broader normative frame within which reliability is 1 condition among several; warranted trust does not follow from reliability alone [51,58]. Fairness, dignity, privacy, transparency, and auditability are therefore treated here not as synonyms for sociotechnical reliability but as cross-cutting constraints on what counts as acceptable AI-enabled care [53,56]. Workflow integration and accountability, in turn, are operational and governance conditions that enable reliability to be achieved and assessed [9,27]. This distinction keeps the construct analytically bounded while recognizing that a technically dependable care pathway may still be unacceptable if it violates fundamental requirements for resident well-being or governance.

## The Closed-Loop Reliability Framework

The proposed framework organizes AI-enabled LTC as a closed-loop safety net comprising 5 interdependent stages: sensing, inference, verification and contextualization, response and escalation, and learning and adaptation. Consistent with sociotechnical approaches that treat AI as 1 component of a larger care system rather than as a stand-alone intervention [9,48], these stages are best understood as 5 reliability functions rather than mandatory sequential steps. Their ordering, salience, and configuration may vary across applications and care contexts; functions may overlap, recur, or operate in parallel as information is interpreted and translated into action. This flexibility is consistent with patient journey and distributed sociotechnical perspectives that emphasize interactions across settings, actors, and system boundaries [43,47]. Governance and accountability operate across all 5 functions rather than constituting a sixth reliability function.

Sensing captures resident- and environment-related information from sources such as wearables, ambient sensors, electronic health records, voice, video, or staff-entered data. Inference transforms these inputs into classifications, predictions, alerts, or recommendations. Verification and contextualization determine whether an AI output is meaningful for the resident in light of their baseline function, cognition, medication, preferences, and goals of care. Response and escalation translate verified information into proportionate care actions, ranging from observation or reassessment to clinical review, family notification, or transfer. Learning and adaptation close the loop by using outcomes, incidents, overrides, drift, and recurrent failures to modify thresholds, workflows, training, or system design.

Figure 1 summarizes the 5-function closed-loop architecture, including feedback relationships and governance and accountability as a cross-cutting layer.

**Figure 1:**
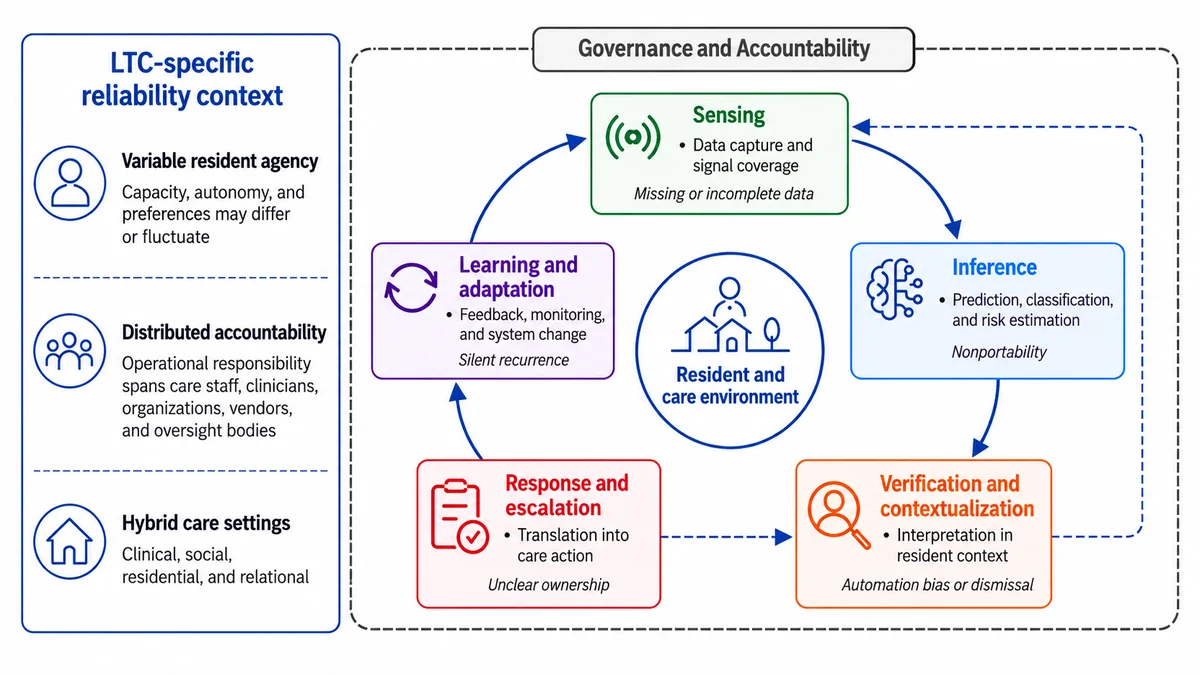
Closed-loop sociotechnical reliability framework for AI-enabled long-term care (LTC). Solid arrows indicate the primary closed-loop pathway, whereas dashed arrows indicate that information may return to earlier functions for additional sensing or reassessment. The framework is functionally organized rather than temporally linear. Governance and accountability are shown as an enclosing cross-cutting layer that operates across all 5 functions rather than forming a sixth function. Italicized text under each function identifies a recurrent failure mode detailed in Table 2. Function labels and structure, rather than color alone, distinguish the 5 functions.

This functional organization differs from a technology-based taxonomy. Existing LTC AI literature commonly distinguishes technologies such as sensors, fall detection systems, predictive models, decision support tools, and care robots [1,4]. In the closed-loop framework, however, these technologies may span several reliability functions: sensors and wearables contribute primarily to sensing; fall detection and predictive alerts span sensing and inference; and decision support or resident-facing systems may extend into verification and response. The purpose is therefore not to assign each technology to a fixed category but to identify where reliability is gained or lost as AI-generated information is translated into care action. Caregiver-facing applications are best understood in this light: not as a separate technology class but as a translation layer at which inference becomes interpretation and interpretation becomes care.

Textbox 1 applies this architecture to fall detection, illustrating how strong algorithmic performance can coexist with failures in verification and contextualization, response and escalation, or learning and adaptation elsewhere in the care pathway.

**When a fall detector is not a safety net: a worked example.**

Fall detection is among the most studied AI-enabled monitoring applications in the care of older adults and is commonly evaluated using device-level performance measures [16,22]. A fall detector is not reliable simply because it detects falls accurately; reliability emerges when the care system converts its signal into timely, contextualized, accountable action and learns from the resulting outcomes. The five reliability functions, described as follows, illustrate how a technically capable system can still fail to deliver reliable care:

1. Sensing: a wearable or ambient sensor captures resident movement. Reliability can fail before inference begins: devices may be removed or discontinued, while missing data, sensor failure, false positives, and false negatives can distort coverage and propagate downstream [14,18,19,22]. The algorithm may therefore operate on an incomplete representation of the resident’s risk state.
2. Inference: the captured signal is classified as a fall or a nonfall event. Model performance may not transfer reliably to long-term care populations, where evidence remains heterogeneous and locally grounded validation is limited [4,10]. False-positive and false-negative signals further illustrate why device-level correctness does not necessarily amount to clinically meaningful inference for an individual resident [16,18].
3. Verification and contextualization: the alert reaches a designated human verifier—here, a nurse or care worker—who interprets it in the context of baseline mobility, recent medication changes, cognition, and goals of care. Two failure models recur: automation bias, in which the output is accepted without sufficient review, and dismissal, in which repeated, poorly aligned alerts erode attention or trust [25,32]. Verification therefore depends on available time, appropriate authority, and resident-specific knowledge.
4. Response and escalation: a verified event must trigger timely and proportionate action, with clarity about who must act, when the action must occur, and what authority is required. Reliability depends on defined workflows, staffing arrangements, role expectations, and escalation pathways [27,31]. An alert without an accountable response pathway is not, by itself, a safety intervention.
5. Learning and adaptation: true falls, missed falls, false alarms, and delayed responses should inform threshold adjustment, training, workflow redesign, and model monitoring [29,30,54]. Governance operates across all 5 functions; learning and adaptation is the function through which monitoring, audit, and review are translated into system change.

## Governance and Accountability as a Cross-Cutting Layer

Learning and adaptation close the loop; governance and accountability are not a final stage or a sixth reliability function. They operate across all 5 reliability functions, setting the conditions under which each is performed and making the pathway accountable and auditable across the AI system’s lifecycle [30,54].

Residents vary widely in capacity, autonomy, and preferences, and their ability to notice, question, or contest AI outputs may fluctuate. Reliability therefore cannot depend on a presumed level of resident participation. Resident involvement should be supported wherever possible, but the system must retain verification, escalation, and accountability mechanisms when direct participation is limited.

First, verification responsibility should be designated in advance. Where human verification is required, a named role—the designated human verifier—should have both the authority and sufficient operational capacity to review, contextualize, override, or escalate the output. The appropriate role depends on the application, setting, risk associated with incorrect action, and authority required for the response [25,32,48]. Second, residents, representatives, and surrogates should have defined routes to question or contest AI outputs and resulting care decisions. These routes strengthen accountability but are not prerequisites for system reliability; where representative participation is unavailable, an alternative verification and escalation pathway must remain operative [58]. Third, escalation and fallback pathways should be auditable. Records of what was detected, who reviewed it, what action followed, and where the pathway failed should be retrievable so that incidents can be traced, reviewed, and translated into learning and adaptation [28,30,54].

These arrangements prospectively allocate operational roles among care staff, clinicians, provider organizations, and technology vendors, while making explicit the participation of residents and representatives and the roles of regulators and other external oversight bodies. They do not determine legal liability, which remains jurisdiction-dependent [58]. The narrower claim is that operational responsibility should be specified before failure rather than reconstructed only after it.

## Scope and Boundary Conditions

The framework applies to AI systems that generate resident-relevant information, predictions, recommendations, or actions that may influence care, safety, or well-being. Its configuration varies by application: monitoring and decision support systems may engage all 5 reliability functions explicitly, whereas socially assistive robots or conversational systems fall within scope when their outputs or actions can affect resident care, safety, or well-being [35,39]. By contrast, systems that do not generate resident-relevant outputs or inform resident-level care decisions—such as rostering, billing, and supply chain systems, or purely administrative documentation tools—fall outside the framework’s scope.

The framework can be applied across institutional LTC, assisted living, home-based LTC, and ageing-in-place settings, but the relevant actors, workflows, resources, and accountability structures differ across these environments [5]. Transferability therefore requires adaptation rather than an assumption of a uniform configuration across settings.

Proportionality should not be defined by a universal numerical threshold. The appropriate response depends on acuity and time sensitivity, signal uncertainty, the consequences and reversibility of acting or delaying action, and the operational capacity actually available. Local governance should specify target verification and response windows according to acuity and the responsible role, together with escalation pathways for circumstances in which those targets cannot be sustained.

Operational capacity is a design constraint, not a justification for accepting greater resident risk. Where staffing or workflow cannot sustainably support required verification and escalation, responses may include prioritization, pathway redesign, additional safeguards, restricted functionality, or, where appropriate, suspension of the relevant use [27,48]. Persistent verification overload should be treated primarily as a system design and governance signal rather than presumptively as individual staff failure. Decisions to restrict or suspend use should be assessed against the realistic alternative care process, not against an assumption of zero risk in the absence of the AI system.

## Failure Modes Across the Care Pathway

### Overview

AI-enabled care can fail even when individual technical components perform as intended. The recurrent failure modes identified across the literature cluster around failures in sensing, inference, verification, response, and learning. The concern here is therefore not to restate what each function does but to identify where the pathway from AI-generated information to care action becomes unreliable.

### Sensing: Incomplete or Distorted Representation

Sensing fails when captured data do not adequately represent the resident, routine, or environment on which the inference is based. Signal loss, false positives, false negatives, obstruction, and sensor failure can propagate directly into misleading inference and inappropriate response [15-18]. Reliability may also degrade through nonuse, partial use, or discontinuation when monitoring conflicts with residents’ routines or preferences [14,20]. Sensor-derived information further depends on clear rules for access, interpretation, and privacy-sensitive use [19,21]. Key reliability conditions are therefore the monitoring of data completeness and missingness, fault detection, context-sensitive coverage, and safeguards against treating incomplete data as an absence of risk.

### Inference: Nonportability, Measurement Error, and Bias

The principal inference risk is that models developed in 1 population or data environment may not transport reliably to LTC residents. Existing decision support evidence remains heterogeneous, with limited validation across LTC applications [4,10]. Differences in frailty, multimorbidity, cognition, polypharmacy, functional dependence, and documentation can affect calibration and clinical meaning. Label and measurement error can further distort prediction, as illustrated by misclassification and reporting delays in nursing home fall documentation [11]. Biased data or deployment practices may also reproduce inequities in prediction or care allocation [53,56]. Reliability therefore requires population-relevant validation, calibration, subgroup assessment, monitoring of data quality, and an explicit linkage between predicted risk and intended care action.

### Verification and Contextualization: Overreliance or Dismissal

Verification can fail in opposite directions. Automation bias may lead users to accept AI outputs without sufficient contextual review [25], whereas repeated false or poorly aligned alerts may lead to routine dismissal and loss of trust [32]. Explainability alone does not resolve this problem: explanations are context-dependent and may themselves create unwarranted confidence [24,55]. In LTC, interpretation must also account for resident-specific baselines, goals, preferences, and care relationships [12,57-59]. Socially interactive technologies illustrate how meaning and trust may be distributed across residents, staff, and families rather than located in the model alone [35,39]. Reliability conditions include resourced verification, resident-specific context, authority to question or override outputs, and documentation of overrides and unresolved uncertainty.

### Response and Escalation: Alert Burden, Ownership, and Delayed Action

A technically valid alert provides little protection if no one owns the response, action is delayed, or escalation pathways are unclear. Excessive or poorly targeted alerts can create fatigue and weaken attention to clinically meaningful signals [26], while postdeployment optimization may require threshold adjustment, severity reclassification, or contextual tailoring [29]. Real-world implementation studies show that response depends on defined workflows, staffing arrangements, role expectations, and escalation pathways [27,31]. Failures therefore include unresolved alerts, fragmented handoffs, responsibility without authority, and disproportionate responses to false-positive or uncertain signals. Reliability requires role-based routing, time-sensitive escalation, handoff standards, and mechanisms for identifying when alert burden exceeds the sustainable operational capacity.

### Learning and Adaptation: Recurrence Without Correction

The loop fails when incidents, false alarms, overrides, model drift, or workflow breakdowns do not lead to system change. Sociotechnical and implementation frameworks emphasize that performance changes as staffing, documentation, workflows, and organizational conditions evolve [41,42,46,49]. Clinical AI literature similarly supports postdeployment monitoring, adverse event review, model maintenance, bias surveillance, and iterative adaptation [28,30,54,60]. Learning and adaptation therefore require the detection of recurrent failures, review of failures and near misses, monitoring for drift and inequity, and completion of corrective actions. Governance determines whether these signals are visible and acted upon, but it remains a cross-cutting layer rather than constituting a sixth reliability function.

Table 2 summarizes these recurrent failure modes, their consequences for residents and care delivery, and the corresponding reliability conditions across the 5 functions.

**Table 2:** Recurrent failure modes and reliability conditions across the 5 reliability functions^a^.

Reliability functions	Recurrent failure modes	Consequences for residents and care delivery	Reliability conditions
Sensing	Missing or incomplete data, sensor failure, and nonuse	Missed or delayed recognition of need, false reassurance, and additional checking	Monitoring of data completeness and missingness, fault detection, and context-sensitive coverage
Inference	Nonportability, misclassification, and biased prediction	Missed or overestimated risk, inequitable care, and alert burden	Long-term care–relevant validation, calibration, subgroup assessment, and linkage of predictions to care actions
Verification and contextualization	Automation bias, alert dismissal, and inadequate resident-specific context	Misinterpreted risk, inappropriate action, loss of trust, and interpretive burden	Resourced verification, resident-specific context, authority to override outputs, and documentation of uncertainty
Response and escalation	Unclear ownership, delayed response, handoff failure, and overload	Delayed assistance, preventable harm, task overload, and responsibility without authority	Role-based routing, time-sensitive escalation, handoff standards, and capacity monitoring
Learning and adaptation	Silent recurrence, model drift, inadequate incident review, and incomplete corrective action	Repeated preventable harm, persistent inequity, and accumulating workaround burden	Incident and drift monitoring, completion of corrective actions, and workflow or model adaptation

^a^Items are illustrative rather than exhaustive and summarize the failure modes discussed in the preceding section. Governance and accountability operate across all 5 reliability functions and do not constitute a sixth function.

## Operationalizing Reliability

The framework is intended to be observable, not merely descriptive. Each reliability function can be interrogated using the 4 evaluation questions set out earlier: where the pathway is failing, who holds the authority to verify an output and act on it, whether the required response is sustainable within available staffing and workflow capacity, and whether failures become visible to governance and feed into learning and adaptation. Table 3 presents illustrative indicators through which these questions can be examined in a given deployment.

**Table 3:** Illustrative indicators and evaluation questions for each reliability function^a^.

Reliability functions	Illustrative indicators	Illustrative evaluation question
Sensing	Data completeness and missingness, device adherence and discontinuation, and coverage of settings and situations in which risk arises	Does the captured signal represent the resident well enough to support action, and can incomplete data be distinguished from an absence of risk?
Inference	Calibration and subgroup performance in the deployed population, false-positive and false-negative rates in practice, drift over time	Does the output carry the clinical meaning it is assumed to carry for the resident group?
Verification and contextualization	Proportion of outputs reaching a designated verifier within a locally specified window, override and dismissal rates, documentation of unresolved uncertainty, routes for residents or representatives to contest outputs	Who holds the authority to verify the output and act on it, and can that verification be sustained with the time and resident-specific knowledge?
Response and escalation	Response latency against locally set targets by acuity, unresolved and unowned alerts, completion of escalation and handoffs, and alert volume relative to available staffing	Can the required response be sustained within available staffing and workflow capacity?
Learning and adaptation	Recurrence of previously identified failures, completion of near-miss and incident review, completion of corrective actions, and auditability of escalation and fallback events	Do failures become visible to governance and inform learning and adaptation?

^a^Indicators and evaluation questions are illustrative rather than validated or prescriptive. Their relevance, thresholds, and feasibility are application- and setting-dependent, and they are not intended to form a composite reliability score. Reviewing the indicators across functions is intended to identify where reliability is being lost along the pathway.

These indicators extend evaluation beyond model metrics to pathway and process measures. They include, where human verification is required, the proportion of outputs that reach a designated verifier within a locally specified window; the availability of routes for residents or their representatives to question or contest AI outputs and resulting care decisions; whether escalation and fallback events are recoverable from records; and whether identified failures result in completed corrective actions. Target verification and response windows should be specified locally according to acuity and the responsible role rather than prescribed universally.

These indicators are illustrative rather than validated. They are not offered as a scored instrument and should not be aggregated into a composite reliability index: aggregation would obscure the function at which a pathway is failing, which is the framework’s intended diagnostic value. Their selection, thresholds, and feasibility require empirical development in specific settings.

This also identifies gaps in LTC-specific evaluation. Verification, response, and learning warrant study as primary objects of inquiry rather than as implementation details, with residents, representatives, and frontline staff involved in defining what counts as reliable care [36,48].

## Limitations

Several limitations should be acknowledged. This Viewpoint develops a conceptual framework rather than reporting a systematic review; it does not estimate effect sizes or rank interventions. The framework has a practice-informed starting point within a single national context rather than being derived from systematically collected empirical data, and the literature mapping that refined it was interpretive rather than an independent empirical test. Coverage across the 5 reliability functions is uneven, with sensing and inference better represented than verification and contextualization, response and escalation, and learning and adaptation; methodological limitations of the search itself are detailed in Multimedia Appendix 1 [63-66]. The framework is anchored primarily in formally institutionalized LTC, although some supporting evidence comes from adjacent home- and community-based settings; its transferability to informal, home-based, and culturally diverse care arrangements therefore requires further examination. Accountability arrangements are jurisdiction-dependent: the framework specifies where operational responsibility should be assigned prospectively, not who bears legal liability in a particular jurisdiction. Finally, the framework is advanced as a proposition for evaluation rather than as a validated model. Its design, implementation, and governance implications are therefore stated concretely as propositions that can be examined and tested in practice.

## Toward a Care System Reliability Paradigm

If reliability is a property of the care system rather than of the model alone, responsibility must extend beyond model validation. Developers should support verification, override, and monitoring; provider organizations should align deployment with sustainable workforce and workflow capacity; and regulators and oversight bodies should address postdeployment auditing, recourse, and conditions for restriction or withdrawal. Residents and their representatives need clear routes to question AI outputs and understand who is accountable when AI outputs inform care. AI in LTC should ultimately be judged not by what algorithms predict but by whether care systems can translate signals into timely, contextually appropriate, and proportionate action, make responsibility visible, and learn from failure while preserving the human foundations of care.

## Appendix

Multimedia Appendix 1 Supplementary literature mapping and framework refinement.

## Abbreviations

LTC: long-term care
NASSS: nonadoption, abandonment, scale-up, spread, and sustainability
SEIPS: Systems Engineering Initiative for Patient Safety

## Generative AI Use

Generative AI assistants, including ChatGPT (GPT-5 series; OpenAI) and Claude (Fable 5; Anthropic PBC), were used to support language editing, restructuring, condensation, formatting, and consistency checking during manuscript preparation and revision. The conceptual framework, scientific arguments, literature selection and interpretation, and substantive content of the tables and figures were developed by the authors. All AI-assisted content and revision decisions were reviewed and approved by the authors, who take full responsibility for the manuscript.
