# Supplementary material for: AI in Long-Term Care: A Closed-Loop Sociotechnical Reliability Framework
Source: J Med Internet Res. 2026 Oct 28;28:e105161. doi: 10.2196/105161 (PMC13755774; doi:10.2196/105161)
Supplement: Supplementary file 1 [file jmir_v28i1e105161_app1.docx]

Supplementary Appendix

AI in Long-Term Care: A Closed-Loop Sociotechnical Reliability Framework

Supplementary literature mapping and framework refinement

S1. Purpose

This appendix describes the purposive literature mapping used to interrogate and refine the framework presented in this Viewpoint. The manuscript is not a systematic review or meta-analysis, and the search was not designed to estimate pooled effects, provide exhaustive coverage of AI applications in long-term care (LTC), or assess the comparative effectiveness.

The five reliability functions were formulated before the mapping, informed by the corresponding author's professional experience overseeing AI initiatives across long-term care and hospital settings. The literature was then examined to determine whether analogous reliability concerns were reported more broadly, to sharpen the content and boundaries of the functions, and to characterize recurrent failure modes and reliability conditions.

The mapping was organized across six-complementary evidence domains selected because each addressed a distinct component of the end-to-end reliability problem. References were mapped to sensing, inference, verification and contextualization, response and escalation, and learning and adaptation; governance and accountability were treated to a cross-cutting rather than as a six function. A single reference could inform more than one domain or function.

The search focused on LTC and adjacent digital-care domains that could inform sensing reliability, inference, human verification, alert governance, workflow integration, response and escalation, organizational capacity, or accountability. Adjacent domains were included when they involved AI-mediated monitoring, support, or decision-making for care-dependent populations and offered conceptually transferable evidence relevant to at least one reliability function. General Telehealth, consumer-facing mHealth applications, and digital therapeutics without passive, ambient, or context-sensitive monitoring were not included as primary evidence domains, although selected papers were used where they clarified specific conceptual or ethical issues.

S2. Databases and sources

PubMed was used as the primary bibliographic database, including MEDLINE-indexed and other PubMed-indexed records available at the time of search. The search was designed to support purposive framework mapping rather than an exhaustive, effect-size-oriented systematic review. PubMed provides broad coverage of clinical, nursing, gerontology, digital health, implementation, ethics, and health-services literature relevant to AI-enabled care systems. To mitigate the limitations of a single-database strategy, the structured searches were supplemented by targeted reference checking of key reviews, conceptual papers, and domain-defining studies.

Reference checking was applied to a predefined set of source types: (a) systematic and scoping reviews identified in each domain search that addressed reliability, implementation, or governance of AI or digital care systems; (b) conceptual and theoretical papers relevant to reliability functions or to the conditions associated with them; and (c) studies repeatedly cited across the retrieved literature, suggesting foundational relevance within their domain. Reference lists of these papers were viewed, and any potentially relevant paper identified was assessed against the same retention criteria applied to database-retrieved records.

Search concepts were developed for each evidence domain using concept blocks. Within each block, synonyms and related terms were combined with OR; across blocks, concept blocks were combined with AND. Where feasible, MeSH terms were combined with free-text title and abstract searching to maintain sensitivity without excessive loss of specificity. In two domains in which core theoretical terms are well defined and domain-specific (sociotechnical systems and responsible AI), title-field restriction was applied to selected concept-block terms to reduce noise from adjacent literatures. The terms shown in Table S1 represent the core search concepts used across domains.

S3. Search period

Database searches were limited to literature published from January 1, 2010, to April 30, 2026. The 2010 starting point was chosen to balance historical coverage with conceptual relevance, and to capture the digital care ecosystem from which contemporary AI-enabled care technologies emerged. This period span the expansion of electronic health record infrastructure [S1], the translation of machine-learning methods into health care [S2], the consolidation of ambient assisted living and smart home technologies for older-adult monitoring [S3], and the subsequent growth of clinical decision support, care robotics, responsible AI, and real-world AI implementation research [S2,S4].

Although AI in long-term care has become prominent more recently, its current possibilities rest on earlier developments in AI methods, digital care infrastructure, and sociotechnical health systems. Restricting the search to more recent literature would have obscured foundational work on ambient sensing reliability, clinical decision support, and sociotechnical systems that inform the interpretation of the framework.

S4. Retention and use in framework mapping

References were retained for mapping if they (a) addressed reliability, failure, safety, or implementation of AI or digital care technologies in LTC or in an adjacent domain meeting the criteria described in S1, and (b) supported, challenged, qualified, or exposed gaps in one or more reliability functions. References were not excluded solely on the basis of study design. Their methodological limitations, setting, population, and conceptual transferability were considered when determining how they informed the framework.

The five reliability functions had been formulated before literature mapping. Retention decisions and refinement of the functions' content and boundaries then proceeded iteratively. Where mapping exposed gaps in the coverage of particular failure modes or reliability conditions, search concepts were refined and targeted follow-up searches or reference checking were undertaken. Mapping was conducted by the first author, who was not involved in the initial formulation of the five reliability functions, and reviewed by the corresponding author, with ambiguous mappings resolved through discussion; it was not performed as independent duplicate screening or coding.

Candidate reliability conditions were retained when they addressed a recognizable failure point in translating AI-generated information into care, were recurrently supported in relevant literature or grounded in an established sociotechnical or responsible-AI principle, and could be expressed as a system-level requirement applicable beyond a single technology or use case. No numerical citation threshold, vote-counting rule, or formal evidence-weighting scheme was applied.

References retained in the framework-mapping matrix constitute the core mapped literature. Other references could be used for contextual or methodological framing without being counted as core mappings, and a single reference could inform more than one evidence domain or reliability function. Search concepts are summarized in Table S1, and the reference-to-framework mappings are provided in Table S4.

S5. Summary of search results by evidence domain-

The six PubMed domain searches retrieved 3,686 domain-level records before deduplication. After deduplication by PubMed Unique Identifier (PMID), these records corresponded to 3,520 unique records. Because the searches were designed to map evidence across overlapping conceptual domains, some records appeared in more than one domain and were coded according to their contribution to the framework rather than treated as mutually exclusive evidence categories.

Domain-level retrievals were retrieved as follows: AI in LTC (n = 491); smart home technologies, wearable sensors, and ambient assisted living (n = 546); clinical decision support and real-world clinical AI (n = 557); care robotics and social robots (n = 385); sociotechnical systems, workflow, and health information infrastructure (n = 856); and responsible AI, ethics, and external accountability (n = 851). These figures precede deduplication and relevance assessment against the criteria described in S5 and Table S3, and are summarized in Table S2. The flow from identification through deduplication and purposive retention to the mapped core reference is shown in Figure S1.

S6. Limitations of the literature mapping

This appendix addresses methodological limitations of the literature mapping; broader conceptual and substantive limitations of the framework itself are discussed in the Limitation section of the main manuscript. The search was purposive rather than exhaustive, and we do not claim to have identified all relevant literature.

First, publication bias may have led to under-representation of implementation failures, abandonment, or negative findings — precisely the evidence most relevant to a reliability-oriented framework. Second, the structured searches were confined to PubMed/MEDLINE. Relevant engineering, computer science, human–computer interaction, and robotics literature indexed primarily in IEEE Xplore, ACM Digital Library, Scopus, or Web of Science may therefore have been missed. This is a particular concern for the smart home and ambient assisted living domain, the care robotics domain, and the sociotechnical systems domain, where technical and human–computer interaction literature is disproportionately indexed outside PubMed/MEDLINE. This limitation was partly mitigated through reference-list checking and selective forward citation checking of key reviews and conceptual papers, as described in S2, but the mapped literature should be read as framework-informing rather than exhaustive. Third, the restriction to English-language sources may have introduced geographic bias and under-represented implementation experiences from non-Anglophone LTC systems, including those in East Asia, continental Europe, and the Global South. Fourth, the exclusion of grey literature, conference proceedings not indexed in PubMed/MEDLINE, and unpublished implementation reports may have produced a lag between real-world AI deployment experiences and the published literature. Fifth, the structured search was not re-run during revision; accordingly, literature published after April 30, 2026 is not represented in the mapped literature. The revision focused on clarifying the framework, its boundaries, and its operational implications rather than updating evidence coverage. Sixth, although the mapping was allowed to refine the content and boundaries of the five reliability functions, it did not constitute an independent empirical test of the framework. The functions preceded the mapping, and the literature was interrogated for support, challenge, qualification, and gaps rather than used to device the literature. Taken together, these limitations mean that the literature mapping should be interpreted as framework-informing rather than exhaustive or validating. Refinements arising from the mapping are conceptual and should not be interpreted as evidence of external validity.

Table S1. Six evidence domains used to inform and refine the framework

| Evidence domain | Focus and key concepts | Role in framework refinement | Primary framework elements informed |
| --- | --- | --- | --- |
| D1. AI in long-term care | LTC-specific AI applications including monitoring, decision support, falls management, care robots, ambient AI, and conversational agents; acceptability, effectiveness, implementation barriers, and responsible innovation in LTC and residential aged care. | Provides LTC-specific application context and identifies recurrent implementation concerns. | Sensing, inference, and response and escalation, with additional contributions to verification and contextualization, learning and adaptation, and normative constraints on acceptable AI-enabled care. |
| D2. Smart home technologies, wearable sensors, and ambient assisted living (AAL) | Passive sensing, wearables, IoT, ambient and contactless monitoring, fall detection, activity recognition; device and signal reliability, sensor failure and fault tolerance, acceptability, privacy, and sustained user adherence in community-dwelling older adults and LTC. | Characterizes sensing technologies, failure modes, and contextual conditions affecting data capture and use. | Primarily sensing and inference, with additional contributions to verification and contextualization, cross-cutting governance/accountability, and normative constraints on acceptable AI-enabled care. |
| D3. Clinical decision support and real-world clinical AI | Predictive alerts, early-warning systems, ML-based risk prediction, and clinical decision support systems (CDSS); automation bias, alert fatigue, override behavior, clinician trust, workflow integration, post-implementation safety, and machine learning operations (MLOps). | Provides adjacent clinical-AI examples of inference, verification, response, monitoring, and governance in real-world care workflows. | Inference, verification and contextualization, response and escalation, and learning and adaptation, with substantial contributions to cross-cutting governance/accountability and normative constraints on acceptable AI-enabled care. |
| D4. Care robotics and social robots | Socially assistive robots (SARs), humanoid robots, companion robots (e.g., PARO, LOVOT), and AI-enabled care robots; human-robot interaction, professional and caregiver acceptance, ethical concerns, and organizational readiness in older-adult and dementia care. | Examines human–robot interaction, care integration, and ethical conditions surrounding socially assistive technologies. | Primarily verification and contextualization, response and escalation, with normative constraints on acceptable AI-enabled care and selected contributions to cross-cutting governance/accountability. |
| D5. Sociotechnical systems and health information infrastructure | Sociotechnical models of health care work systems, including SEIPS, SEIPS 2.0, and SEIPS 3.0; NPT; the NASSS framework; resilience engineering; and AI-specific sociotechnical and normative extensions. | Provides system-level concepts for distributed work, adaptation, resilience, and technology-enabled care across settings. | Predominantly all five reliability functions, with additional contributions to learning and adaptation, response and escalation, and selected cross-cutting governance/accountability and normative constraints on acceptable AI-enabled care. |
| D6. Responsible AI, ethics, and external accountability | Trustworthy AI; algorithmic fairness, bias, and health equity; transparency, explainability and its limits; AI governance frameworks; lifecycle and post-deployment oversight; legal and ethical considerations for AI in post-acute and long-term care; older-adult perspectives on explainable AI. | Examines normative, governance, fairness, explainability, and accountability conditions across AI development and use. | Primarily cross-cutting governance/accountability and normative constraints on acceptable AI-enabled care, with additional contributions to verification and contextualization, inference, and learning and adaptation; one reference maps across all five reliability functions. |

Note: The six evidence domains were selected for their complementary conceptual contributions to the framework rather than to provide exhaustive coverage of the literature. The “Primary framework elements informed” column summarizes the principal contribution of each domain; individual references could inform more than one reliability function or non-function annotation. Detailed reference-to-framework mappings are provided in Table S5.

Abbreviations: AAL = ambient assisted living; AI = artificial intelligence; CDSS = clinical decision support systems; IoT = Internet of things; LTC = long-term care; ML = machine learning; MLOps = machine learning operations; NASSS = non-adoption, abandonment, scale-up, spread, and sustainability; NPT = normalization process theory; PARO = Personal Assistive RObot; SAR = socially assistive robot; SEIPS = Systems Engineering Initiative for Patient Safety.

| Table S2. Domain-specific search concepts used for purposive literature mapping | | | |
| --- | --- | --- | --- |
| Evidence domain | Concept block 1: Technology or system | Concept block 2: Setting or population | Concept block 3: Reliability, implementation, or governance relevance |
| D1. AI in long-term care | "artificial intelligence"; AI; "machine learning"; "deep learning"; "predictive analytics"; "AI-enabled"; "AI-assisted"; algorithm; "decision support"; "fall detection"; "fall prevention"; "resident monitoring" | "long-term care"; "long term care"; "nursing home*"; "residential care"; "aged care"; "assisted living"; "dementia care"; "older adults" | acceptability; effectiveness; implementation; "responsible innovation"; safety; "decision support"; reliability; failure; barriers; facilitators |
| D2. Smart home, wearable sensors, and ambient assisted living | "smart home*"; "ambient assisted living"; AAL; "wearable*"; sensor*; IoT; "internet of things"; "passive monitoring"; "ambient monitoring"; "fall detection"; "activity recognition"; "in-home positioning"; contactless | "older adults"; elderly; "aging in place"; "ageing in place"; "long-term care"; "nursing home*"; "community-dwelling"; frailty; dementia | reliability; "false alarm*"; "false positive*"; "missing data"; "sensor failure"; "fault tolerance"; acceptability; privacy; adherence; usability; governance |
| D3. Clinical decision support and real-world clinical AI | "clinical decision support"; CDSS; CDS; "early warning"; "predictive alert*"; "machine learning"; "deep learning"; "predictive model*"; "risk prediction"; "AI-enabled"; "machine learning operations"; MLOps | hospital; clinical; healthcare; "real world"; "real-world"; deployment; implementation; "post-implementation" | "automation bias"; "alert fatigue"; "override*"; trust; "human oversight"; "clinician override"; "human-AI interaction"; workflow; safety; "post-implementation"; governance |
| D4. Care robotics and social robots | "socially assistive robot*"; SAR; "social robot*"; "humanoid robot*"; "companion robot*"; PARO; LOVOT; "care robot*"; "AI-enabled robot*" | "older adults"; elderly; "long-term care"; "nursing home*"; "dementia care"; "aged care"; "residential care" | acceptance; "human-robot interaction"; HRI; ethics; implementation; barriers; facilitators; "organizational readiness"; caregiver* |
| D5. Sociotechnical systems and health information infrastructure | SEIPS[Title]; "sociotechnical[Title]"; "socio-technical[Title]"; "normalization process theory"; NPT[Title]; NASSS[Title]; "non-adoption"; "resilience engineering"; "human factors"; "distributed cognition"; "situation awareness"; "safety culture" | healthcare; "health care"; clinical; "long-term care"; nursing; hospital; care; "patient safety" | reliability; safety; implementation; "work system"; workflow; "human-AI"; "human-machine"; teaming; governance; accountability |
| D6. Responsible AI, ethics, and external accountability | "responsible AI"[Title]; "trustworthy AI"[Title]; "AI ethics"[Title]; "AI governance"[Title]; "AI fairness"; "explainable AI"[Title]; XAI[Title]; "AI accountability"; "AI bias"; "AI regulation"; "lifecycle" | healthcare; "health care"; clinical; medical; "long-term care"; "older adults"; ageing; aging | fairness; transparency; explainability; accountability; governance; consent; recourse; equity; oversight; lifecycle; "post-deployment" |
| Note: This table summarizes the core search concepts used for each evidence domain (D1–D6). Within each block, terms were combined with OR; across blocks, concept blocks were combined with AND. Where feasible, MeSH terms were combined with free-text searches of titles and abstracts. For evidence domains with well-defined, domain-specific theoretical terms (D5 and D6), title-field restrictions were applied to selected terms within the relevant concept blocks to reduce noise from adjacent literatures.  Search concepts were refined iteratively during literature mapping as the content and boundaries of the reliability functions were sharpened. Refinements included: (i) adding fall-related terms and "resident monitoring" to D1; (ii) adding "false alarm", "false positive", "missing data", and "sensor failure" to D2, corresponding to refinement of the sensing function; (iii) adding "human oversight", "clinician override", and "human-AI interaction" to D3, corresponding to refinement of verification and contextualization; and (iv) adding "resilience engineering", "safety culture", "distributed cognition", and "situation awareness" to D5.  Abbreviations: AAL = ambient assisted living; AI = artificial intelligence; CDSS = clinical decision support systems; HRI = human–robot interaction; IoT = internet of things; LTC = long-term care; MeSH = medical subject headings; MLOps = machine learning operations; NASSS = non-adoption, abandonment, scale-up, spread, and sustainability; NPT = normalization process theory; SAR = socially assistive robot; SEIPS = Systems Engineering Initiative for Patient Safety; XAI = explainable artificial intelligence. | | | |

Table S3. Per-domain PubMed/MEDLINE search results and references retained for framework mapping

| Evidence  domain | Focus | Domain-level records retrieved | References retained for domain mapping |
| --- | --- | --- | --- |
| D1 | AI in long-term care | 491 | 8 |
| D2 | Smart home, wearable sensors, AAL | 546 | 10 |
| D3 | Clinical decision support and real-world clinical AI | 557 | 10 |
| D4 | Care robotics and social robots | 385 | 7 |
| D5 | Sociotechnical systems and health information infrastructure | 856 | 12 |
| D6 | Responsible AI, ethics, and external accountability | 851 | 11 |
| Total | Domain-level records before deduplication | 3,686 | 58 |

Note: Domain-level records were retrieved through structured PubMed/MEDLINE searches completed on April 30, 2026. The retrieval counts precede PMID-based deduplication and relevance assessment against the criteria described in S4 and Table S4. References were retained purposively when they informed the framework by supporting, challenging, qualifying, or exposing gaps in one or more reliability functions, as described in S4. Because the six evidence domains overlap conceptually, a reference could be retained in more than one domain. The total of 58 represents domain-coded mapping entries rather than a count of unique references; these correspond to 57 unique references, one of which is mapped in two domains. A further five references cited in the main text informed contextual or methodological framing without being mapped, as described in S4.

Abbreviations: AAL = ambient assisted living; AI = artificial intelligence; PMID = PubMed Unique Identifier.

Table S4. Relevance criteria applied to retrieved records

| Criterion | Description |
| --- | --- |
| Criteria supporting retention (I) | |
| I1 | LTC or closely related older-adult residential care setting, or community/home-based care involving care-dependent older adults, dementia, frailty, or technologies conceptually transferable to LTC care delivery. |
| I2 | AI-enabled, machine-learning-based, predictive, algorithmic, decision-support, sensing, monitoring, robotic, or ambient/passive digital system used in LTC settings or explicitly applicable to LTC care delivery. |
| I3 | Addresses at least one reliability function of the closed-loop framework (sensing, inference, verification/contextualization, response/escalation, or learning and adaptation), or a cross-cutting governance/accountability or normative condition relevant to those functions. |
| I4 | Addresses at least one reliability-relevant issue, including safety, failure modes, missing data, false alarms, alert fatigue, workflow integration, human oversight, implementation, usability, privacy, bias, accountability, ethics, or governance. |
| Criteria supporting non-retention (E) | |
| E1 | Studies conducted exclusively in acute hospital, emergency department, surgical, rehabilitation, sports, or pediatric settings without conceptual transferability to LTC. |
| E2 | Studies of AI applications without a sensing, monitoring, decision-support, robotic, or care-delivery component (e.g., basic image-classification benchmarking, drug-discovery pipelines, algorithm-only studies). |
| E3 | Studies addressing only AI methods, model architecture, or algorithm performance without reference to clinical or care-delivery context, implementation, safety, or governance. |
| E4 | Editorials, commentaries, news items, conference abstracts without methodological detail, study protocols without data, and non-peer-reviewed sources. |
| E5 | Articles not available in English. |
| Note: Criteria were applied to records retrieved from the structured PubMed/MEDLINE domain searches. Assessment focused on conceptual relevance to the reliability framework rather than effect size or study-design hierarchy. Criterion I3 is presented here using the final terminology of the refined framework; the underlying relevance principle remained whether a record informed the care pathway, its cross-cutting governance/accountability conditions, or normative constraints on acceptable AI-enabled care. | |

Figure S1. Practice-informed framework development and purposive literature mapping

Note: The five reliability functions were formulated before literature mapping and were subsequently refined against it. Refinement included, for example, expansion of sensing to include missingness and sensor failure, and of verification and contextualization to include oversight, override, and human–AI interaction. Counts refer to domain-coded mapping entries rather than unique references, since a reference could be retained in more than one domain. Search concepts are given in Table S2, relevance criteria in Table S4, and reference-to-framework mappings in Table S5.

Abbreviations: PMID = PubMed Unique Identifier.

| Table S5. Reference-to-framework mapping matrix across six evidence domains | | | |
| --- | --- | --- | --- |
| no | Reference | Framework stages informed | Key contribution to the framework |
| D1. AI in Long-Term Care (n = 8): LTC application context | | | |
| D1-1 | Loveys et al. Lancet Healthy Longev 2022 [1] | Sensing; Response and escalation | Systematic review of AI-enhanced interventions for older people in LTC, covering acceptability and effectiveness. Reports mixed effectiveness, generally favorable acceptability, and high risk of bias across studies, indicating that the LTC evidence remains developmental. |
| D1-2 | Seibert et al. J Med Internet Res 2021 [13] | Sensing; Inference; Response and escalation | Rapid review mapping AI application scenarios in nursing care, spanning monitoring, activity classification, fall detection, care coordination, documentation, and safety. Shows nursing homes to be under-represented relative to hospitals. |
| D1-3 | Lapp et al. J Med Internet Res 2022 [4] | Inference | Scoping review of decision-support tools in adult LTC across medication management, falls prevention, hospitalization, malnutrition, urinary tract infection, and COVID-19. Identifies limited validation, heterogeneity, and unclear intervention definitions. |
| D1-4 | Lukkien et al. Gerontologist 2023 [5] | Normative constraints on acceptable AI-enabled care | Scoping review of practical approaches to responsible AI in LTC. Identifies user-oriented innovation, context sensitivity, and the framing of AI as a response to innovation challenges, and reports limited empirical detail on local LTC contexts. |
| D1-5 | Lukkien et al. JMIR Nurs 2024 [12] | Verification and contextualization; Learning and adaptation; Normative constraints on acceptable AI-enabled care | Qualitative study of prerequisites for the responsible embedding of AI-based decision support in LTC. Identifies deliberation over data collection, balanced proactive support, trust-aligned incremental implementation, customization, bias mitigation, human-centered learning loops, and routinization. Human-centered learning loops involve care staff in design and use, including iterative refinement of system logic and outputs in response to new data and user feedback. |
| D1-6 | Charon et al. Stud Health Technol Inform 2022 [10] | Inference | Develops an explainable Bayesian-network classifier for pressure-ulcer prediction using nursing-home electronic medical records, reporting better performance than existing nursing-home scores. |
| D1-7 | Graham et al. J Am Med Dir Assoc 2026 [11] | Sensing; Inference | Applies natural language processing to LTC electronic health record free text for fall documentation, reporting 96% accuracy against chart review. Identifies under-reporting, misclassification, and reporting delay in the Minimum Data Set, indicating potential to improve the sensitivity and temporal precision of fall documentation. |
| D1-8 | Neves et al. J Appl Gerontol 2023 [6] | Normative constraints on acceptable AI-enabled care | Qualitative analysis of how AI for later life is imagined, designed, deployed, and resisted. Introduces the concepts of promissory discourse and ageing anxieties to account for sociotechnical ageism in LTC AI. |
| D2. Smart Home, Wearable Sensors, and Ambient Assisted Living (n = 10): Sensing and contextual conditions | | | |
| D2-1 | Denecke et al. Yearb Med Inform 2021 [17] | Sensing. | Examines safety of ambient assisted living technologies and distinguishes user safety from system safety. Identifies system reliability, data quality, and sensor integration as conditions of safe operation, and notes the absence of agreed safety metrics for AAL. |
| D2-2 | ElHady et al. Sensors (Basel) 2018 [18] | Sensing; inference | Systematic survey of sensor failure detection and fault tolerance in ambient assisted living. Shows how sensor failure, false positives, and false negatives propagate into misleading downstream classification. |
| D2-3 | Chan et al. JMIR Aging 2024 [15] | Sensing; inference | Systematic review of in-home localization for older adults, including ambient sensors used in the context of frailty, dementia, and ageing in place. Reports how position and movement are derived from sensor data. |
| D2-4 | Chaudhuri et al. J Geriatr Phys Ther 2014 [16] | Sensing | Systematic review of fall detection devices used with older adults, covering device accuracy, passive monitoring, real-world testing, and acceptability. Reports false alarms and missed detections and highlights limitations in real-world testing. |
| D2-5 | Stavropoulos et al. Sensors (Basel) 2020 [22] | Sensing; inference | Literature review of Internet of Things wearables and smart-home devices in eldercare, covering monitoring, fall detection, indoor positioning, assessment, accuracy, and acceptability. Describes how sensor data are used for monitoring and risk assessment. |
| D2-6 | Wang et al. Internet Things 2025 [23] | Sensing; inference | Compares contactless AI-driven approaches to recognizing activities of daily living in terms of unobtrusiveness, contextual richness, scalability, and real-world deployment, with privacy discussed as a design consideration. Notes that larger and more diverse datasets are needed. |
| D2-7 | Moore et al. JMIR Mhealth Uhealth 2021 [20] | Sensing | Qualitative systematic review and meta-synthesis of older adults' experience of long-term wearable use. Identifies motivation, usability, device purpose, support structures, and integration into daily life as determinants of sustained use, and reports discontinuation as a common outcome. |
| D2-8 | Pirzada et al. Inform Health Soc Care 2022 [21] | Sensing; Cross-cutting governance/accountability; Normative constraints on acceptable AI-enabled care | Reviews ethical and acceptability challenges in smart homes for older adults, including privacy, social support, autonomy, usability, training, dignity, and independence, and discusses user control over monitoring and ownership of sensor-generated data. |
| D2-9 | Berridge et al. Ageing Soc 2017 [14] | Sensing; Normative constraints on acceptable AI-enabled care | Resident-centered account of responses to a passive monitoring system in low-income independent living, including adoption, non-adoption, discontinuation, creative misuse, and disruption of daily routines. Reports resident concerns related to autonomy and control alongside patterns of use and non-use. |
| D2-10 | Hunter et al. JMIR Mhealth Uhealth 2020 [19] | Verification and contextualization; Cross-cutting governance/accountability | Focus-group study with health care professionals on the management and governance of sensor data. Identifies who may access sensor data, how data are interpreted across professional roles, and the institutional conditions for acting on sensor-derived information. |
| D3. Clinical Decision Support and Real-World Clinical AI (n = 10): Clinical workflow and post-deployment conditions | | | |
| D3-1 | Goddard et al. J Am Med Inform Assoc 2012 [25] | Verification and contextualization | Systematic review of automation bias, defining over-reliance and insufficient monitoring of automated output. Identifies training, accountability, display design, and confidence information as mitigating factors. |
| D3-2 | Amann et al. BMC Med Inform Decis Mak 2020 [24] | Verification and contextualization; Cross-cutting governance/accountability; Normative constraints on acceptable AI-enabled care | Multidisciplinary analysis of explainability in health care across technical, medical, legal, ethical, and patient perspectives. Identifies informed consent, certification and approval, and liability as legal considerations, and examines implications for ethical values and clinical adoption. |
| D3-3 | Sandhu et al. J Med Internet Res 2020 [31] | Inference; Verification and contextualization; Response and escalation | Qualitative study of how a machine-learning sepsis early-warning system became embedded in emergency department workflows. Describes how defined processes and workforce arrangements shaped whether model outputs were interpreted and acted on. |
| D3-4 | Joshi et al. JAMIA Open 2022 [27] | Inference; Response and escalation | Qualitative study comparing implementation of rule-based and machine-learning sepsis risk prediction. Reports organizational and workflow barriers to the clinical use of model outputs. |
| D3-5 | van der Vegt et al. J Am Med Inform Assoc 2023 [33] | Inference; Response and escalation; Learning and adaptation; Cross-cutting governance/accountability; Normative constraints on acceptable AI-enabled care | Systematic review of deployed machine-learning sepsis prediction algorithms, mapping implementation barriers, enablers, and decision points to the SALIENT end-to-end clinical AI implementation framework. Identifies clinical workflow, evaluation and monitoring, multidisciplinary governance, and ethics-related considerations, including surveillance-bias monitoring and model explainability, across implementation. |
| D3-6 | Schwartz et al. JMIR Hum Factors 2022 [32] | Verification and contextualization; Response and escalation | Qualitative descriptive study of clinician trust in predictive clinical decision support for in-hospital deterioration. Identifies understandability, perceived accuracy, and clinician role as factors shaping trust, and reports that trust influenced whether deterioration alerts were acted on. |
| D3-7 | Graafsma et al. J Am Med Inform Assoc 2024 [26] | Inference; Response and escalation | Scoping review of AI-based methods for optimizing medication alerts generated by clinical decision support systems. Reports alert fatigue and limited clinical value of alerts, and a gap between statistical model performance and measured clinical outcomes. |
| D3-8 | Ledger et al. J Am Med Inform Assoc 2023 [29] | Response and escalation; Learning and adaptation; Cross-cutting governance/accountability | Scoping review of post-implementation optimization of medication alerts, covering alert inactivation, severity reclassification, contextual tailoring, and threshold changes. Reports that included studies evaluated alert quantity and acceptance after modification and describes multidisciplinary governance structures for alert oversight. |
| D3-9 | Labkoff et al. J Am Med Inform Assoc 2024 [28] | Verification and contextualization; Learning and adaptation; Cross-cutting governance/accountability; Normative constraints on acceptable AI-enabled care | Consensus-based recommendations for AI-enabled clinical decision support addressing trustworthiness, bias mitigation, transparency, and safety. Specifies validation, verification, and certification processes, performance monitoring, adverse-event reporting, pre- and post-deployment evaluation, and rules and guidelines for oversight. |
| D3-10 | Rajagopal et al. Mayo Clin Proc Digit Health 2024 [30] | Inference; Learning and adaptation; Cross-cutting governance/accountability | Scoping review of machine learning operations in health care. Describes model monitoring and performance, automated retraining, clinical workflow integration, deployment infrastructure, and regulatory considerations across the machine-learning lifecycle. |
| D4. Care Robotics and Social Robots (n = 7): Human–robot interaction and care integration conditions | | | |
| D4-1 | Lee YH et al. J Med Internet Res 2025 [37] | Verification and contextualization; Response and escalation | Systematic review of health care professionals' perspectives on socially assistive robots in health care settings. Identifies acceptance, preparedness, trust, and ethical considerations as factors affecting whether robots are integrated into care activities. |
| D4-2 | Papadopoulos et al. BMJ Open 2020 [39] | Verification and contextualization; Response and escalation | Systematic review of enablers and barriers to implementing socially assistive humanoid robots in health and social care, restricted to studies involving hands-on interaction. Reports user-, professional-, organizational-, and technology-related factors affecting implementation and use. |
| D4-3 | Hung et al. BMC Geriatr 2019 [35] | Verification and contextualization; Response and escalation; Normative constraints on acceptable AI-enabled care | Scoping review of the social robot PARO in care settings, including dementia care and long-term care. Documents clinical and behavioral effects, acceptance across settings, and barriers including cost, workload, infection concerns, stigma, and ethical issues. |
| D4-4 | Franke et al. Front Psychol 2021 [34] | Verification and contextualization; Normative constraints on acceptable AI-enabled care | Theoretical paper proposing a three-stage assessment model based on acceptance, ethics, and emotions for implementing assistive technologies and robotics in long-term care facilities. Addresses organizational culture, staff and leadership emotions, and ethical norms. |
| D4-5 | Leineweber et al. Med Health Care Philos 2026 [38] | Verification and contextualization; Cross-cutting governance/accountability; Normative constraints on acceptable AI-enabled care | Systematic qualitative review of ethical aspects of social robot use in caring for older people. Identifies concerns involving dignity, autonomy, deception, privacy, and accountability in the use and deployment of social robots. |
| D4-6 | Shi et al. Int J Nurs Stud 2025 [40] | Verification and contextualization; Response and escalation | Systematic review of qualitative studies on caregivers' perceptions and experiences of socially assistive robots in dementia care. Reports how caregiver trust shaped interpretation of robot behavior and how caregivers described robots as supporting care-related tasks. |
| D4-7 | Lee K et al. Digit Health 2025 [36] | Verification and contextualization; Normative constraints on acceptable AI-enabled care | Critical reflection co-authored with people living with dementia and family care partners on experiences of an AI-enabled socially assistive robot in long-term care. Reports engagement and conversational support alongside concerns about human touch, emotional nuance, ownership, equitable allocation, cultural responsiveness, and safety. |
| D5. Sociotechnical Systems and Health Information Infrastructure (n = 12): Sociotechnical and adaptive system conditions | | | |
| D5-1 | Carayon et al. Appl Ergon 2020 [43] | All five reliability functions | Extends the SEIPS model to the patient journey, framing safety as distributed across time, settings, actors, and organizational boundaries rather than located at a single point of care. |
| D5-2 | Carayon et al. Appl Ergon 2014 [42] | All five reliability functions | Human factors systems approach defining health care quality and patient safety as properties of the work system rather than of individual components, arising from interactions among people, tasks, tools, environments, and organizations. |
| D5-3 | Holden et al. Ergonomics 2013 [45] | All five reliability functions | Extends SEIPS to account for configuration, engagement, and adaptation within work systems. Describes context-specific arrangements among work-system elements, participation of patients and staff in care processes, and changes in those arrangements over time. |
| D5-4 | Sittig et al. Qual Saf Health Care 2010 [49] | All five reliability functions | Eight-dimensional sociotechnical model for health information technology, covering hardware and software, clinical content, human–computer interface, people, workflow and communication, internal organizational policies, external rules and regulations, and system measurement and monitoring. |
| D5-5 | Salwei et al. J Cogn Eng Decis Mak 2022 [48] | All five reliability functions | Sociotechnical systems framework for applying AI in health care delivery, positioning AI as one element of a larger work system and examining its interactions with people, tasks, technologies, organizational conditions, and clinical workflow. |
| D5-6 | McCradden et al. Patterns (NY) 2023 [9] | All five reliability functions; Cross-cutting governance/accountability; Normative constraints on acceptable AI-enabled care | Normative framework treating AI as an intervention ensemble rather than a stand-alone tool and setting out how such an ensemble should be appraised in relation to the legitimate interests of patients and the health system. Addresses institutional oversight and regulation alongside responsible and ethical appraisal, evaluation, and use. |
| D5-7 | Murray et al. BMC Med 2010 [46] | Learning and adaptation | Normalization process theory as a framework for developing, evaluating, and implementing complex interventions, with four constructs: coherence, cognitive participation, collective action, and reflexive monitoring. Addresses embedding into routine practice and appraisal leading to reconfiguration of the intervention. |
| D5-8 | Naikar et al. Ergonomics 2023 [47] | All five reliability functions | Critiques dyadic models of human–automation interaction and argues for distributed, joint, and self-organizing perspectives in which system performance emerges from interactions among multiple human and technological agents within changing work contexts. |
| D5-9 | Abimbola et al. BMC Med 2019 [41] | Learning and adaptation | Applies the NASSS framework retrospectively to a technology-supported change program, analyzing emergent uncertainties, interdependencies, and patchy uptake and sustainability as the program unfolded over time. |
| D5-10 | Fairbanks et al. Jt Comm J Qual Patient Saf 2014 [44] | Response and escalation; Learning and adaptation | Reviews resilience and resilience engineering in health care, framing safety as adaptive capacity rather than the absence of failure, and covering how systems fail safely, recover, and learn from both adverse and successful events. |
| D5-11 | Greenhalgh et al. J Med Internet Res 2017 [8] | All five reliability functions | Framework for theorizing and evaluating non-adoption, abandonment, and challenges to scale-up, spread, and sustainability, covering condition, technology, value proposition, adopter system, organization, wider context, and adaptation over time. |
| D5-12 | Sutton et al. NPJ Digit Med 2020 [50] | Inference; Verification and contextualization; Response and escalation | Overview of clinical decision support systems, covering how patient-specific data and clinical knowledge generate recommendations, how clinicians assess and override alerts, and how alert fatigue and workflow disruption affect whether clinically important alerts lead to action. |
| D6. Responsible AI, Ethics, and External Accountability (n = 11): Normative and governance conditions | | | |
| D6-1 | Anderson B. Nat Med 2024 [51] | Cross-cutting governance/accountability | Commentary on the relationship between AI innovation and regulatory oversight in health care, framing innovation and regulation as complementary rather than opposed requirements for safe deployment. |
| D6-2 | Solaiman et al. J Am Med Dir Assoc 2024 [58] | Verification and contextualization; Cross-cutting governance/accountability; Normative constraints on acceptable AI-enabled care | Legal and ethical analysis of AI use for residents in post-acute and long-term care, using residents' social, psychological, and mobility needs as a context-specific gauge for benefits and risks. Examines autonomy, algorithmic discrimination, privacy and data use, and consent, including concerns arising with cognitive decline. |
| D6-3 | Rubeis G. Arch Gerontol Geriatr 2020 [57] | Normative constraints on acceptable AI-enabled care | Ethical analysis of AI-based gerontechnology in older-adult care, conceptualizing risks as depersonalization, discrimination, dehumanization, and disciplining through monitoring and surveillance. Outlines patient-centered strategies to preserve autonomy, dignity, participation, and relational care. |
| D6-4 | Char et al. N Engl J Med 2018 [52] | Inference; Verification and contextualization; Cross-cutting governance/accountability; Normative constraints on acceptable AI-enabled care | Analysis of ethical challenges in implementing machine learning in health care, moving from abstract principles toward implementation. Examines bias in prediction, the role of human judgement, accountability, and integration into clinical care. |
| D6-5 | Amann et al. BMC Med Inform Decis Mak 2020 [24] | Verification and contextualization; Cross-cutting governance/accountability; Normative constraints on acceptable AI-enabled care | Multidisciplinary analysis of explainability in health care across technical, medical, legal, ethical, and patient perspectives. Identifies informed consent, certification and approval, and liability as legal considerations, and examines implications for ethical values and clinical adoption. |
| D6-6 | Ghassemi et al. Lancet Digit Health 2021 [55] | Verification and contextualization; Normative constraints on acceptable AI-enabled care | Cautions against assuming that current explainable AI methods produce trust, transparency, or bias mitigation for patient-level decision support. Describes failure cases and argues for rigorous internal and external validation rather than reliance on explanation alone. |
| D6-7 | Rajkomar et al. Ann Intern Med 2018 [56] | Inference; Cross-cutting governance/accountability; Normative constraints on acceptable AI-enabled care | Analysis of fairness in machine learning for health, describing how model design, historical data, and interactions with clinicians and patients can produce unequal predictions, outcomes, and resource allocation. Proposes fairness criteria across design, deployment, and evaluation and makes policy recommendations specifying stakeholder roles, responsibilities, and oversight. |
| D6-8 | Chen et al. Annu Rev Biomed Data Sci 2021 [53] | All five reliability functions; Cross-cutting governance/accountability; Normative constraints on acceptable AI-enabled care | Review of ethical machine learning in health care organized around a pipeline from problem selection through data collection, model development, evaluation, deployment, and post-deployment considerations. Examines equity and bias across the pipeline, including post-deployment auditing and regulatory considerations. |
| D6-9 | Zhang et al. BMC Med Inform Decis Mak 2023 [60] | Verification and contextualization; Cross-cutting governance/accountability; Normative constraints on acceptable AI-enabled care | Examines trustworthiness in medical AI through data quality, algorithmic bias, opacity, safety and security, and responsibility attribution. Proposes ethical, legal, and regulatory countermeasures including transparency and traceability, whole-process review, and risk monitoring and control. |
| D6-10 | Collins et al. JAMIA Open 2024 [54] | Learning and adaptation; Normative constraints on acceptable AI-enabled care | Applies a lifecycle-informed approach to ethical review of health care AI, treating development, use, and refinement as iterative and recognizing that stakeholders may identify different ethical issues at different lifecycle points. Covers development, deployment, monitoring, refinement, and decommissioning. |
| D6-11 | van Leersum et al. Gerontologist 2026 [59] | Verification and contextualization | Empirical interview study of older adults' views on explainable AI in the context of ageing in place. Reports heterogeneous preferences for explanation and identifies joint human–AI decision-making as important to many participants. |
| Note: The table contains 58 domain-coded mapping entries across six evidence domains, corresponding to 57 unique references; because a reference could inform more than one domain, entries do not represent 58 separate references. Amann et al. [24] is mapped in both D3 and D6. Mappings use the five reliability functions together with two non-function annotations. Cross-cutting governance/accountability denotes operational and governance conditions through which responsibility is assigned, audited, and enforced, and is represented as the enclosing layer in Figure 1. Normative constraints on acceptable AI-enabled care denote fairness, dignity, privacy, transparency, auditability, and related requirements that constrain what counts as an acceptable way of achieving reliability; they do not constitute a second structural layer or an additional reliability function. Bracketed numbers refer to the reference list of the main manuscript. Mappings indicate conceptual contribution to framework refinement and do not imply evidence ranking or empirical validation. | | | |

**References**

S1 Blumenthal D, Tavenner M. The “Meaningful Use” Regulation for Electronic Health Records. N Engl J Med. 2010;363:501-504.

S2 Jiang F, Jiang Y, Zhi H, et al. Artificial intelligence in healthcare: past, present and future. Stroke Vasc Neurol. 2017;2:230-243.

S3 Rashidi P, Mihailidis A. A survey on ambient-assisted living tools for older adults. IEEE J Biomed Health Inform. 2013;17:579-590.

S4 Topol EJ. High-performance medicine: the convergence of human and artificial intelligence. Nat Med. 2019;25:44-56.
